# Role of hypoxia inducible factor-1 in keratinocyte inflammatory response and neutrophil recruitment

**DOI:** 10.1186/1476-9255-10-28

**Published:** 2013-08-10

**Authors:** Emma Leire, Josh Olson, Hart Isaacs, Victor Nizet, Andrew Hollands

**Affiliations:** 1 Department of Pediatrics, University of California, San Diego, La Jolla, CA, USA; 2 School of Pharmaceutical Sciences, University of Copenhagen, Copenhagen, Denmark; 3 Rady Children’s Hospital, San Diego, California, USA; 4 Skaggs School of Pharmacy and Pharmaceutical Sciences, University of California, San Diego, La Jolla, CA, USA; 5 University of Queensland, Brisbane, QLD, Australia

**Keywords:** Hypoxia-inducible factor, HIF, Cytokine, Keratinocyte, Neutrophil recruitment, Inflammation

## Abstract

**Background:**

Hypoxia inducible factor-1 (HIF-1) is a major regulator of the cellular adaption to low oxygen stress and the innate immune function of myeloid cells. Treatment with the novel HIF-1 stabilizing drug AKB-4924 has been shown to enhance the bactericidal activity of keratinocytes as well as phagocytic cells. In this study, we sought to investigate the effect of pharmacological boosting of HIF-1 with AKB-4924 in keratinocytes and their contribution to the innate immune response.

**Findings:**

Treatment with the novel HIF-1 stabilizing drug AKB-4924 can increase keratinocyte production of pro-inflammatory cytokines *in vitro* and enhance neutrophil recruitment *in vivo*.

**Conclusions:**

HIF plays an important role in cytokine production by keratinocytes and in neutrophil recruitment to the skin. The HIF-boosting drug AKB-4924 has the potential to enhance the immune response even in the complex environment of bacterial skin infections.

## Introduction

Hypoxia inducible factor-1 (HIF-1) is a transcription factor that is a major regulator of cellular adaption to low oxygen levels [1,2]. More recently, HIF has been recognized to play a role in host defense, promoting the bactericidal activity of phagocytic and epithelial cells and supporting innate immune activities of dendritic cells and mast cells [3-6]. HIF is comprised of a constitutively expressed β subunit that partners with dynamically regulated α subunits (HIF-1α, HIF-2α, or HIF-3α), which are unstable under normoxic conditions [7]. The short half-life of HIF-α subunits reflects the activity of a family of oxygen-and iron-dependent prolyl hydroxylases (PHDs), which direct HIF-α subunits to proteosomal degradation [8]. A new PHD inhibitor, AKB-4924, acts as a HIF-1α stabilizing agent to increase HIF-1 levels and boosts the bactericidal activity of cultured phagocytes and skin cells against important bacterial pathogens [9]. Furthermore, local administration of AKB-4924 *in vivo* was shown to limit progression of *Staphylococcus aureus* skin infection [9]. AKB-4924 has previously been shown to stabilize HIF-1α, and to a lesser degree HIF-2α, in mouse fibroblasts, demonstrated by up-regulation of known HIF target genes such as vascular endothelial growth factor (VEGF) and glucose transporter-1 [9]. However, the effects of AKB-4924 on a cellular level in keratinocytes that contribute to the observed innate immune boosting remain unclear. In this focused study, we investigated the effect of the HIF-stabilizing agent AKB-4924 on keratinocytes. Specifically, the production of VEGF, involved in enhancing permeability of the local microvasculature [10], interleukin-8 (IL-8), a C-X-C family chemokine that is the major neutrophil chemo-attractant in humans [11] and interleukin-6 (IL-6), a pro-inflammatory cytokine that supports neutrophil antibacterial activity in the skin, and the role that HIF plays in neutrophil recruitment *in vivo*[12].

## Methods

### AKB-4924

AKB-4924 was manufactured as previously described in a three-step synthesis with a resulting purity of 98% [9]. Structure and purity were confirmed by nuclear magnetic resonance followed by mass spectrometry. AKB-4924 was resuspended in dimethyl sulfoxide, pH = 4.2-4.4 to 5 mM and used at 10 μM unless otherwise indicated [9].

### Cell culture

HaCaT cells (ATCC), a human skin keratinocyte cell line [13], were plated in 12 well plates in RPMI supplemented with 10% fetal bovine serum (FBS) and penicillin/streptomycin (Invitrogen) and grown to confluency. The cells were washed with PBS and fresh media was added containing 10 μM AKB-4924, and/or 1 μg/mL LPS (Sigma) or vehicle control. Cells were incubated in normoxia (37°C, 5% CO_2,_ 21% O_2_) or hypoxia (37°C, 5% CO_2,_ 1% O_2_) for 24 h in a hypoxia chamber.

### Measurement of cytokine production

Following culture under the conditions described above, supernatant was collected and concentration of IL-6, IL-8 and VEGF was measured by enzyme linked immunosorbent assay (ELISA) as per the manufacturer’s instructions (R&D Systems) and expressed as pg/ml.

For *in vivo* cytokine analysis wildtype (WT) and knockout (KO) mice were injected intradermally with 5 μg LPS in 100 μl PBS. After 24 h, skin was collected from euthanized animals using a 10 mm biopsy punch (Miltex) and homogenized as described above. Levels of mouse IL-6 (mIL-6), mouse VEGF (mVEGF) and the mouse IL-8 homologue keratinocyte-derived cytokine (KC) in skin homogenate were determined by ELISA as per the manufacturer’s directions (R&D). Data were expressed as pg/wound.

### HIF-1 keratinocyte knockout mice

Keratinocyte-specific inactivation of HIF-1α was achieved by cross-breeding K14-cre transgenic mice [14] with HIF-1α flox/flox mice [15]. In all experiments, cre-negative littermates served as WT controls.

### Neutrophil recruitment assays

For neutrophil recruitment studies in WT and KO mice, animals were administered intradermal injections with 5 μg LPS in 100 μl PBS, or PBS alone as a control, into the shaved skin of the flank. Each mouse received two LPS injections and two PBS only injections. Mice were sacrificed after 24 h and skin was harvested from the site of injection using a 10 mm biopsy punch (Miltex). One skin sample per condition from each mouse was fixed in 10% formalin and stained with hematoxylin and eosin (H&E) by the UCSD histology core facility. Histology was scored as follows: 0 = no inflammation, 1 = mild inflammation, 2 = moderate inflammation, 3 = severe inflammation. The other skin sample of each condition was resuspended in PBS and homogenized as previously described [16]. Neutrophil migration was quantified by a myeloperoxidase (MPO) assay [17]. To enumerate neutrophils in mouse skin homogenates, a standard curve with human neutrophil derived myeloperoxidase was used to quantify MPO. Human neutrophils were collected by venipuncture from healthy volunteers and harvested using PolyMorphPrep according to the manufacturer’s protocol (Axis-Shield, Norway). For HIF boosting studies utilizing AKB-4924, CD1 outbred mice were administered intradermal injections with 1 μg LPS in 100 μl PBS, or PBS alone as a control, into the shaved skin of the flank as described above, with or without 100 μM AKB-4924.

### Statistical analyses

Statistical analyses were performed using the Prism software from GraphPad (La Jolla, CA). All data were expressed as mean ± standard deviation and analyzed using Student’s unpaired *t*-test or one-way ANOVA.

### Ethics approvals

Permission to collect human blood under informed consent was approved by the UCSD Human Research Protections Program. All animal experiments were conducted according to the guidelines approved by the UCSD Institutional Animal Care and Use Committee.

## Results

### Effect of HIF boosting on cytokine production in keratinocytes

**Figure 1:**
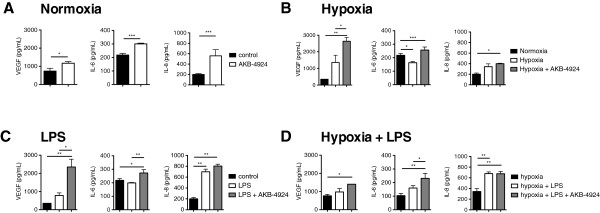
**AKB-4924 boosts production of VEGF, IL-6 and IL-8 by HaCat keratinocytes *in vitro. *A** Cytokine production in control cells after the addition of vehicle (black bars) or after the addition of 10 μM AKB-4924 (white bars). **B** Cytokine production under normoxia (black bars), hypoxia (white bars), or hypoxia with AKB-4924 (gray bars). **C** Cytokine production in control cells (black bars), cells stimulated with LPS (white bars), or cells treated with LPS in conjunction with AKB-4924 (gray bars). **D** Cytokine production by cells under hypoxia alone (black bars), LPS stimulation under hypoxia (white bars), or LPS stimulation in conjunction with AKB-4924 under hypoxia (gray bars). For all panels, * indicates *P* < 0.05, ** indicates *P* < 0.01, *** indicates *P* < 0.001 as determined by student’s *t*-test or one-way ANOVA.

In this study we investigated the effect of HIF boosting on keratinocyte cytokine production, and we found that keratinocytes treated with the HIF-1 stabilizer AKB-4924 produced significantly more VEGF, IL-6 and IL-8 compared to cells treated with vehicle alone (Figure 1A).

### HIF boosting in keratinocytes under hypoxic conditions

Skin is hypoxic under baseline conditions, and tissue and wounds have even lower oxygen levels [5]. To examine the effect of pharmacological stabilization of HIF-1 under hypoxic conditions, we treated keratinocytes with AKB-4924 or vehicle control in a hypoxic (1% O_2_) environment for 24 h. While a modest up-regulation of IL-8 and VEGF under hypoxia compared to normoxia did not achieve statistical significance, and a significant decrease in IL-6 was seen during hypoxia compared to normoxia, hypoxia plus the administration of AKB-4924 resulted in significant increases in VEGF, IL-6 and IL-8 (Figure 1B). A significant increase in VEGF and IL-6 were also observed in hypoxia plus AKB-4924 compared to hypoxia alone.

### HIF boosting in keratinocytes in conjunction with an inflammatory stimulus

To investigate the effects of HIF boosting by AKB-4924 in keratinocytes under the influence of an inflammatory stimulus, cells were treated with 1 μg/mL LPS and AKB-4924 or vehicle control for 24 h under normoxic conditions. IL-8, but not IL-6 production, was significantly increased in response to LPS alone (Figure 1C). A modest, non-significant increase of VEGF was observed in keratinocytes treated with LPS. However, concurrent treatment of LPS plus AKB-4924 resulted in significantly higher levels of VEGF, IL-6, and IL-8 compared to control cells. VEGF and IL-6 production were also significantly higher in cells stimulated with LPS in conjunction with AKB-4924 treatment compared to cells stimulated with LPS alone. Treatment with AKB-4924 did not significantly increase IL-8 levels above those of LPS treatment alone, suggesting that this level of LPS stimulation results in already maximal IL-8 expression.

### HIF boosting in keratinocytes in conjunction with an inflammatory stimulus under hypoxic conditions

To more closely mimic the hypoxic conditions of an infected skin lesion, keratinocytes were stimulated with 1 μg/mL LPS, with or without the addition of AKB-4924, in 1% O_2_. Similar to normoxic conditions, IL-8 but not VEGF or IL-6 production was significantly increased in response to LPS under hypoxic conditions (Figure 1D). VEGF, IL-6 and IL-8 were all significantly higher after stimulation with LPS in conjunction with AKB-4924 compared to control cells, suggesting that AKB-4924 can further boost HIF-regulated cytokine production beyond that supported by hypoxia alone. As seen with LPS stimulation alone, IL-8 levels were not significantly increased with AKB-4924 treatment compared to LPS in hypoxia without AKB-4924.

### HIF boosting with AKB-4924 enhances neutrophil recruitment *in vivo*

**Figure 2:**
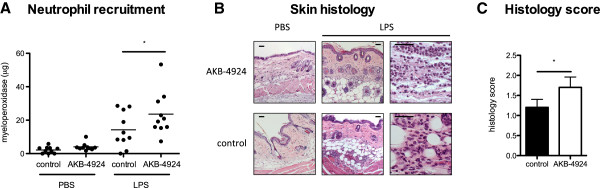
**AKB**-**4924 enhances neutrophil recruitment *in vivo. *A** Neutrophil recruitment to the site of LPS injection as determined by MPO assay. **B** H&E stained section of mouse skin injected with LPS and AKB-4924 or LPS and vehicle control, 24 h post-injection in low and high power magnification. Scale bars = 50 µm. **C** Histological scoring of H&E stained skin sections. * indicates *P* < 0.05.

To determine the *in vivo* effect of enhanced chemokine and VEGF production by HIF-boosted keratinocytes on neutrophil recruitment, we injected 1 μg LPS intradermally into the flanks of WT mice, with or without AKB-4924. Neutrophil recruitment was determined by quantifying MPO in tissue homogenates 24 h after LPS treatment. AKB-4924 significantly increased the recruitment of neutrophils to the site of LPS injection (Figure 2A). Histological analysis confirmed an inflammatory response consisting of primarily neutrophils, with some macrophages and lymphocytes also present (Figure 2B). No difference was observed in the type of cells recruited, but an overall increase in inflammatory response was seen in AKB-4924 treated animals, as shown by histological score (Figure 2C).

### HIF-1α in keratinocytes contributes to neutrophil recruitment and cytokine production *in vivo*

**Figure 3:**
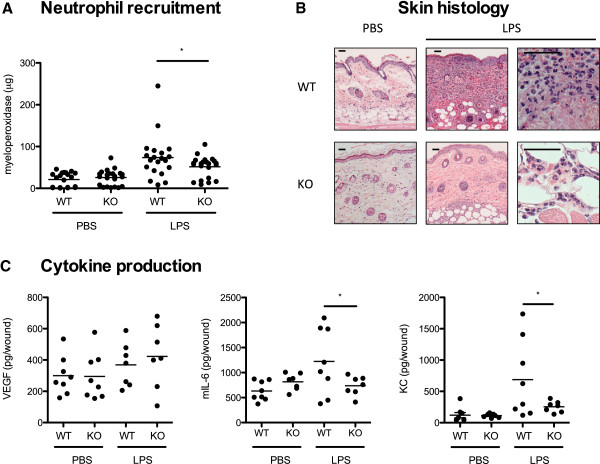
**HIF-1α KO mice have reduced response to LPS *in vivo. *A** Neutrophil recruitment to the site of LPS injection as determined by MPO assay. **B** H&E stained section of mouse skin injected with LPS, 24 h post-injection in low and high power magnification. Scale bars = 50 µm. **C** Amount of mVEGF, mIL-6, and KC in mouse skin, 24 h post-injection with LPS. * indicates *P* < 0.05.

Mice with specific deletion of HIF-1α in keratinocytes have previously been shown to develop significantly larger necrotic skin lesions with higher bacterial load compared to WT mice [5]. To study the role of HIF-1α in keratinocytes in recruitment of neutrophils to an inflammatory stimulus, WT and keratinocyte-specific HIF-1α KO mice were injected intradermally with a high dose (5 μg) of LPS. Significantly fewer neutrophils were recruited to the site of injection in HIF-1α KO mice compared to WT controls (Figure 3A). Histological analysis confirmed a strong inflammatory response in WT mice (Figure 3B). No differences were observed in the cell types recruited in WT and KO animals, with the inflammatory response consisting primarily of neutrophils at this timepoint.

To determine whether HIF-1α in keratinocytes plays an essential role in cytokine production *in vivo*, WT and KO mice were injected intradermally with LPS and 24 h post-injection, skin tissue was homogenized and used for ELISA. At this time point no difference in mVEGF was observed between WT and KO mice, however significantly lower levels of mIL-6 and the murine IL-8 homologue KC were produced in KO mice (Figure 3C). No significant difference in levels of mVEGF, mIL-6, or KC was observed between WT and KO mock (PBS) treated animals.

## Discussion

In this study we investigated specific effects of the HIF-boosting agent AKB-4924 in keratinocytes in tissue culture conditions mimicking those found *in vivo* including hypoxia and exposure to toll like receptor (TLR) agonist, LPS. In addition, we investigated HIF-boosting with AKB-4924 *in vivo*. Under most circumstances, AKB-4924 significantly increased keratinocyte production of VEGF, IL-6, and IL-8 (Figure 1), which are known to contribute to neutrophil migration and activation, vascular permeability, wound healing and skin cell proliferation. While a decrease in IL-6 was observed in hypoxia compared to normoxia (Figure 1B), treatment with AKB-4924 not only reversed this decrease in IL-6 but also resulted in a significant increase in IL-6 levels compared to normoxia. This finding suggests that additional cellular changes during the shift to hypoxic conditions, and not HIF levels alone, can contribute to altered cytokine production in keratinocytes.

Our data showed that AKB-4924 is capable of boosting keratinocyte production of cytokines involved in the inflammatory response beyond that of immune stimuli alone under both hypoxia and normoxia. IL-8 levels were not increased with AKB-4924 beyond those seen with LPS stimulation in normoxia or hypoxia suggesting that IL-8 may already be maximally expressed in keratinocytes exposed to the TLR agonist LPS. These findings suggest that AKB-4924 has the potential to further boost the keratinocyte response supporting neutrophil recruitment, even in an environment where inflammatory stimuli and hypoxia are already acting to stimulate HIF pathways, as corroborated *in vivo* in Figure 2 with analysis of MPO levels in mouse skin and histological observation. While it is not currently known whether HIF has a direct effect on MPO production by neutrophils, a correlation between MPO in mouse skin samples and histological score of inflammation was observed (Figure 2).

This enhancement of neutrophil recruitment likely contributes to the observed ability of local AKB-4924 administration to enhance bacterial clearance in mouse skin infections, synergizing with increases in specific keratinocyte bactericidal effectors such as cathelicidin peptides [9], and direct effects of the HIF-boosting drug to activate the antimicrobial capacity of the neutrophils following their recruitment.

Mice lacking HIF-1α in their keratinocytes have previously been shown to develop larger necrotic lesions and had decreased capacity to clear bacteria [5]. Here we found that HIF-1α keratinocyte KO mice recruited less neutrophils to the site of administration of an inflammatory stimulus (LPS) compared to WT mice, while producing less mIL-6 and KC than their WT counterparts (Figure 3). This finding demonstrates that while other cell types may contribute to cytokine production in the skin, HIF-1α in keratinocytes plays an important role in recruitment of neutrophils. This delay in the kinetics of neutrophil recruitment resulting from a reduced chemokine response in keratinocytes further explains the innate immune defect in the HIF-1α keratinocyte KO mouse. Previous work with *S*. *aureus* bacterial challenge has shown that even in keratinocyte HIF-1α deficiency, the expanding necrotic lesion is ultimately replete with neutrophils at later time points [5].

Together, these findings support the potential of AKB-4924 to boost keratinocyte innate immune responses that promote neutrophil recruitment to inflammatory stimuli. Coupled with their ability to stimulate myeloid cell bactericidal function [3,9,18], HIF-boosting compounds such as AKB-4924 may represent a useful adjunctive therapy for bacterial infections in an age of ever increasing resistance to front-line antibiotic agents.

## Competing interests

The authors declare that they have no competing interests.

## Authors’ contributions

EL designed and performed *in vitro* and *in vivo* experiments. JO performed *in vivo* experiments. HI interpreted experimental results and performed histological analyses. VN helped conceive of the study and design experiments. AH conceived of the study, designed and performed experiments. EL, VN, and AH wrote the manuscript. All authors read and approved the final manuscript.
